# Jean Martin Charcot

**Published:** 1894-01-06

**Authors:** 


### Two Notable Doctors.

### JEAN MARTIN CHARCOT.

In the summer that has just passed away there died Jean Martin Charcot, at the comparatively early age of sixty-eight. Though born in 1825, it was not until 1853, at the age of twenty-eight, that Charcot took his medical degree. In 1862 he was appointed physician to the Salpétrière Hospital, and it was there that most of his brilliant work was done. Two things made Charcot famous—first, his practical sympathy with his patients and his business-like aptitude for putting them in the way of comfort and cure; and secondly, his ardent love of vital, as distinguished from mere morbid, pathology. It was Charcot's great ambition as a physician to realise in imagination those disease processes which duller minds never perceive until they see them completed on the post-mortem-table. Charcot, with a humanity at once worthier and more scientific, strove to keep his patient from a too intimate acquaintance with the post-mortem table for as long a period as possible. "There can be little doubt," says the *Journal of Pathology*, "that Charcot's teaching on the subjects to which, when Professor of Pathological Anatomy in the Medical Faculty of Paris, he devoted his attention, had a very marked influence on the study of pathology in this country, especially in its bearing on the association of altered structure and modified function. Pathology was with him not merely morbid anatomy, but a perfect, if intricate, association of cause and effect. The researches carried on by him and by his pupils under his guidance bulk largely in the contemporary literature bearing on the pathology of the nervous system. . . . We in this country have come to look upon Charcot as peculiarly at one with us in our method of studying disease. With all the Gallic lucidity and power of exposition—a power sometimes so wanting in the greatest observers—he combined great tenacity of purpose, an almost inexhaustible fund of patience, and an accuracy of definition such as is possessed by few." Charcot was, indeed, a great man of the modern type. He set himself to conquer a portion of the world of science by the modern method, the method, that is to say, of observation, sympathy, and intelligent interpretation—a method as far removed from the older and more vulgar methods of conquest by destruction and extermination as light is from darkness.

